# A randomized pharmacological fMRI trial investigating d-cycloserine and brain plasticity mechanisms in learned pain responses

**DOI:** 10.1038/s41598-022-23769-7

**Published:** 2022-11-09

**Authors:** Mia A. Thomaidou, Joseph S. Blythe, Dieuwke S. Veldhuijzen, Kaya J. Peerdeman, Johan (Hans) P. A. van Lennep, Erik J. Giltay, Henk R. Cremers, Andrea W. M. Evers

**Affiliations:** 1 Faculty of Social and Behavioral Sciences, Leiden University, Wassenaarseweg 52, 2333 AK Leiden, The Netherlands; 2 Leiden Institute for Brain & Cognition, 2333 ZA Leiden, The Netherlands; 3 Department of Psychiatry, Leiden University Medical Center, 2333 ZA Leiden, The Netherlands; 4 Faculty of Social and Behavioral Sciences, University of Amsterdam, 1001 NK Amsterdam, The Netherlands; 5 Medical Delta Healthy Society, Leiden University, Technical University Delft, & Erasmus University Rotterdam, Rotterdam, The Netherlands

**Keywords:** Sensory processing, Randomized controlled trials, Human behaviour, Perception, Clinical pharmacology, Learning and memory, Classical conditioning, Extinction

## Abstract

Learning and negative outcome expectations can increase pain sensitivity, a phenomenon known as nocebo hyperalgesia. Here, we examined how a targeted pharmacological manipulation of learning would impact nocebo responses and their brain correlates. Participants received either a placebo (*n* = 27) or a single 80 mg dose of d-cycloserine (a partial NMDA receptor agonist; *n* = 23) and underwent fMRI. Behavioral conditioning and negative suggestions were used to induce nocebo responses. Participants underwent pre-conditioning outside the scanner. During scanning, we first delivered baseline pain stimulations, followed by nocebo acquisition and extinction phases. During acquisition, high intensity thermal pain was paired with supposed activation of sham electrical stimuli (nocebo trials), whereas moderate pain was administered with inactive electrical stimulation (control trials). Nocebo hyperalgesia was induced in both groups (*p* < 0.001). Nocebo magnitudes and brain activations did not show significant differences between d-cycloserine and placebo. In acquisition and extinction, there were significantly increased activations bilaterally in the amygdala, ACC, and insula, during nocebo compared to control trials. Nocebo acquisition trials also showed increased vlPFC activation. Increased opercular activation differentiated nocebo-augmented pain aggravation from baseline pain. These results support the involvement of integrative cognitive-emotional processes in nocebo hyperalgesia.

## Introduction

Pain can arise as a debilitating symptom that is malleable and highly susceptible to an individual’s internal and external environment^1,2^. Outcome expectations are shown to play a role in shaping pain responses to a given event or treatment^3–5^. While positive outcome expectations can produce beneficial effects from inert treatments (placebo effects), negative outcome expectations can blunt the effect of active interventions and even increase pain sensitivity in response to inert treatments, a phenomenon termed nocebo hyperalgesia^6–9^.

An important process proposed to be involved in nocebo effects is associative learning^10–13^. Classical conditioning is used in experimental nocebo models to form expectations through associative learning^11^. In nocebo conditioning, negative associations form by pairing an inert nocebo stimulus (a sham treatment) to surreptitiously increased pain stimulations. After repeated trials, the nocebo stimulus evokes increases in perceived pain. Negative suggestions are commonly used to enhance conditioning^9,10,14^. Concurrently, conditioned nocebo effects have been shown to effectively reduce using extinction paradigms in which learned associations are discontinued^14–16^. Such paradigms can be adapted for use in MRI settings, using thermal pain stimulations paired with sham electrodes to serve as the nocebo manipulation in conditioning paradigms.

One of the major neural components mediating associative learning processes are the *N*-methyl-d-aspartate (NMDA) receptors^17,18^ whose agonism has been found to augment learning^19–22^. Enhanced NMDA receptor activity promotes local neuroplasticity, which in turn is believed to enhance the acquisition and consolidation of learned material in both animals^23,24^ and humans^25,26^. Studies that used pharmacological agents such as d-cycloserine (DCS) to enhance NMDA-dependent learning support an implication of NMDA receptors in associative learning^27,28^. DCS is a compound that impacts NMDA-mediated neuroplasticity differently in different doses. In lower doses (in most studies varying between 50 and 250 mg) it acts as a partial agonist at the glycine modulatory site of NMDA receptors^29^. To our knowledge, no studies have examined the role of NMDA-mediated learning in nocebo effects.

Findings on extinction-learning and exposure therapy indicate that DCS may be a promising agent for augmenting NMDA-dependent learning^27,30,31^. DCS has also been shown to enhance performance on declarative learning^32^ and generalization of conditioned effects to novel contexts^33^. Studies show that DCS enhances the extinction of phobias and other symptoms resulting from aversive learning^29,34^. Learning studies have shown that DCS can facilitate procedural learning^35^, and extinction or memory consolidation^24,27^. This evidence suggests that by agonizing NMDA receptors, DCS enhances specific learning processes, and can be used to manipulate and investigate how particular learning mechanisms may be involved in pain effects. Previous studies on experimentally induced nocebo and placebo effects indicate an involvement of brain areas that integrate prior experiences and memory into the processing of pain, such as the insula and amygdala^36–40^. Yet, neuroimaging findings in the field of nocebo and learning are still limited and somewhat inconsistent^36^. By utilizing fMRI while pharmacologically agonizing NMDA-mediated learning during nocebo induction, precise neural processes involved in learned pain can be examined.

In the present study we aim to investigate for the first time the role of NMDA-receptor dependent learning in the acquisition and extinction of nocebo effects. Detailed hypotheses and planned analyses were listed in the study pre-registration. Briefly, as compared to placebo administration, we hypothesize that DCS will augment the acquisition of nocebo hyperalgesia and will induce nocebo effects that are more resistant to extinction. We further hypothesize that differential brain activation will be detected between the DCS and placebo groups during nocebo acquisition, evocation, and extinction, in a number of a priori regions of interest such as the prefrontal cortex, anterior cingulate cortex, amygdala, and hippocampus, that were implicated in previous nocebo studies^36^. We also hypothesize that neural activation will differ between the experience of nocebo-augmented pain and the experience of pain stimulations of the same high intensity.

## Materials and methods

### Experimental design

This randomized controlled trial utilizes a placebo-controlled, double-blind design with respect to the pharmacological administration. A double-blind randomization list was created by the Leiden University Medical Center (LUMC) pharmacy. Participants were randomly allocated into blocks of one of two pharmacological groups: DCS or placebo. The random allocation sequence was thus carried out by a party independent from the study and the study investigators enrolled participants and assigned them to the double-blinded pharmacological group. All participants underwent nocebo pre-conditioning outside the scanner and acquisition/extinction procedures in the MR scanner, by use of conditioning and negative verbal suggestions. The entire study consisted of two parts in the same testing day. The screening part lasted approximately 1 h and took place at the department of Social and Behavioral Sciences, Leiden University, the Netherlands. The fMRI part lasted approximately 3 h, of which approximately 1 h took place in the 3 Tesla MRI scanner of the Leiden Institute of Brain and Cognition (LIBC) scanning facilities at the LUMC. This study was pre-registered on ClinicalTrials.gov (NCT04762836; trial registration date 21/02/2021), approved by the Medical Ethics Committee Leiden, The Hague, Delft (P19.003; trial approval date 29/06/2020), and all experiments were performed in accordance with relevant guidelines and regulations for research on human subjects.

### Participants

The required sample size for the primary analysis was calculated based on a previous imaging study that, similar to our primary study objective, investigated the effects of DCS in a learning task^32^. The analysis was conducted in G*power 3.1^41^ for a mixed model analysis of variance (ANOVA). In the experiment by Onur et al.^32^ an ANOVA revealed a main effect of the pharmacological agent (DCS vs. placebo) [*F*(1,27) = 5.454; *P* = 0.027] on performance in a declarative learning task. We derived partial *η*^2^ from the *F* statistic and degrees of freedom^42,43^. With an effect size of *η*^2^ = 0.17, alpha error probability set at α = 0.05, and desired power set at 0.9, the sample size indicated 22 participants per pharmacological group. Given the potential for dropout and artefacts in imaging data, we recruited 25 participants per group in this study.

Inclusion criteria were: age between 18 and 35 years, a good command of the English language, and (corrected to) normal vision and hearing. Exclusion criteria were any history of chronic pain, serious medical or psychiatric conditions, experiencing pain on the day of the study or use of analgesic medication in the 24 h prior to testing, use of psychotropic drugs in the month prior to testing, and being pregnant or breastfeeding. A physician performed a brief health screening based on our exclusion criteria and to assess vital signs. Participants also needed to be eligible to undergo MRI and were screened for standard MRI-compatibility exclusion criteria. Participants were recruited via the recruitment website Sona (Sona Systems, Tallinn, Estonia) and were only able to sign-up for participation if they had not previously participated in similar studies. All participants signed written informed consent and were reimbursed with a 90-euro payment.

### Thermal pain stimulation

Thermal pain stimuli were delivered to participants’ right volar forearm and pain intensities were rated on a numeric rating scale (NRS) ranging from 0 (no pain) to 10 (worst pain imaginable on the arm). In the screening part, pain stimuli were delivered via a Thermal Sensory Analyzer with a 3 × 3 cm thermode probe (TSA-II; Medoc Advanced Medical Systems, Ramat Yishai, Israel). In the MRI part, pain was delivered with an MR-compatible ATS 3 × 3 thermode attached to a Pathway device (Medoc Advanced Medical Systems, Ramat Yishai, Israel).

#### Sensory thresholds

We followed a sensory-thresholds method that follows published standardized and protocolled procedures^44^. To test warmth and pain threshold levels, heat stimuli were applied from a baseline of 32 °C on the forearm and participants were asked to indicate the first moment that they perceived warmth and the first moment that they perceived pain. After a practice trial for each, the average of 3 warmth and 3 pain detection values were calculated as thresholds for warmth and pain, respectively.

#### Pain calibration and administered stimuli

Throughout the experiment, each stimulus was initiated from a 32 °C baseline, increased to a target temperature with ramp up and return rates of 8 °C per second, and presented at peak temperature for 5 s. The maximum temperature that could be reached was 50 °C. The inter-stimulus interval consisted of a pain rating screen with a 6 s duration followed by a fixation cross with a mean duration of 5 s, jittered around a normal distribution of + /− 2 s. Pain calibrations were conducted to select the temperatures that would induce moderate and high pain during nocebo conditioning. The calibrations were individually tailored, based on participants’ NRS ratings of maximum 30 pain stimuli of varying intensities. We used the median temperatures that participants consistently rated as NRS 6–9 (high pain) for nocebo trials in the pre-conditioning and acquisition phases. We used median temperatures consistently rated as approximately NRS 3–5 (moderate pain) for all control trials as well as evocation/extinction nocebo trials.

After calibrations, a nocebo pre-conditioning took place and included 7 nocebo and 7 control trials, to increase the time of learning and ensure nocebo effects would be induced. At the start of the MRI session a baseline phase took place during which 5 high and 5 moderate pain stimuli were administered. The acquisition and extinction phases each included 14 nocebo and 14 control trials. All trials were administered in pseudorandom order, so that no more than three trials of the same type were administered in a row. To reduce habituation or sensitization to heat-pain, we moved the thermode higher on the arm between functional scans; the thermode was moved to a more proximal site on the same arm after baseline and at one third and two thirds of the acquisition/extinction procedure).

### Nocebo manipulation

A commercial Transcutaneous Electrical Nerve Stimulation (TENS) device (Beurer EM 80) was used to deliver (sham) electrical stimuli, which served as the nocebo manipulation in the procedure. Negative verbal suggestions were used to create expectations regarding the pain-enhancing effects of administering electrical stimuli in combination with thermal pain. Two electrodes were placed in a diagonal line on the base of the thumb and the inner elbow. Participants underwent a short mock calibration procedure during which they felt a light electrical pulse through the electrodes (ConMed MR-compatible Cleartrace ECG electrodes). This pulse was delivered in order to increase the believability of the nocebo manipulation. The device was not actually present during conditioning in the MRI scanner, but messages displayed on a computer monitor via E-Prime 3.0 (Psychology Software Tools, Pittsburgh, PA, USA) indicated the sham activation of the electrical stimulation during nocebo trials. Negative suggestions indicated to all participants that when the messages “on” (nocebo stimulus in either purple or yellow font, counterbalanced) and “off” (control stimulus in grey font) were displayed, their pain would be respectively aggravated (nocebo trials) or not altered (control trials). In the pre-conditioning and acquisition phases, the activation of sham electrical stimulation was paired to covertly increased pain stimulation during nocebo trials.

### Pharmacological manipulation

A single dose of DCS was administered at 80 mg for all participants in the DCS group. The LUMC pharmacy prepared DCS (powder form) into capsules, as well as placebo capsules of identical appearance containing the inert agent microcrystalline cellulose. Because plasma concentrations were expected to peak between 1 and 3 h after DCS administration^45,46^, participants ingested the capsule 2 h before entering the MRI scanner to undergo the main learning paradigm.

### Measures

#### Pain

Throughout the experiment, participants were provided with a 6-s window to rate their pain on a sliding scale representing the pain NRS, following each pain stimulation. A message, presented on the computer monitor 2 s after the pain stimulus returned to skin temperature, prompted the pain rating to be given by use of a keyboard in the screening part and button boxes in the MRI session.

#### Learning

To assess learning rates before and after the administration of DCS, participants completed the Wechsler Memory Scale–Fourth Edition (WMS–IV) subtest Verbal Paired Associates^47^. The test was performed twice, once before the administration of DCS and once at the end of the scanning session.

#### MRI

Data were collected at the Leiden Institute for Brain and Cognition imaging facilities at the Leiden University Medical Center, using a Phillips Achieva 3 Tesla scanner with a maximum gradient strength of 40 mT/m, bore diameter of 60 cm, and field-of-view of 45 cm (head-feet direction), and a 32-channel head coil. A structural MRI was made with a T1 weighted gradient echo sequence. Functional scans were taken utilizing T2* weighted gradient echo planar images (TR = 2200 ms, voxel size = 2.75 × 2.5 × 2.75 mm, TE = 30 ms, flip angle = 80°, matrix = 80 × 80, field of view = 220 × 220, slice thickness = 2.75 mm, slice gap = 0.28 mm, 40 slices per volume, sensitivity encoding factor = 2) with a 32-channel SENSE head coil. Functional scans began with two automatically discarded dummy volumes to allow for magnetic field stabilization. Heart rate (finger pulse oximeter) and respiration (respiratory belt transducer) were measured to correct for physiological artifacts during scanning.

#### Questionnaires

A questionnaire containing demographic and health questions was used to screen participants for inclusion. An MRI-compatibility questionnaire was also used, to ensure participants were eligible to enter the scanner. The Structured Clinical Interview for the Diagnostic and Statistical manual for Mental disorders (SCID-5-RV^48^ was used to screen participants for psychiatric disorders. The following four questionnaires were used to measure psychological characteristics: a short version of the State-Trait Anxiety Inventory, State version (STAI-S–s)^49,50^, the State-Trait Anxiety Inventory, Trait version (STAI-T)^50^, the Pain Catastrophizing Scale (PCS)^51^ which assesses catastrophizing thoughts or worrying relating to pain^52^, and the Body Vigilance Scale (BVS)^53^ which measures vigilance about bodily sensations. Total scores were used for all questionnaires.

Participants also completed an exit questionnaire at the end of the experiment, containing manipulation checks and questions about their participation and side effects. The questions were: “did you believe the information you received in this study”, “how much did you worry about what the experimenter thought of you or changing your responses to please them”, “were you focused on the pain stimulations during the study”, and “did you notice the association between the electrical stimuli and pain aggravation”. Questions were rated on a 0–10 NRS from “not at all” to “very much”. All questionnaires were displayed on a computer monitor via web-based survey software (Qualtrics, Provo, Utah, USA).

### Study procedures

**Figure 1:**
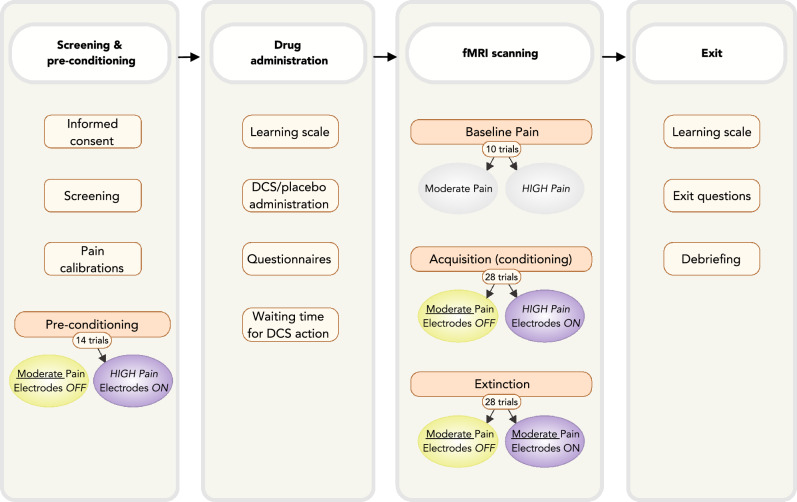
Timeline of the experimental procedure of this study. After screening for inclusion and pain calibrations, participants underwent a pre-conditioning phase. After the first learning task, D-cycloserine (DCS) or placebo was administered, and the psychological questionnaires were completed. In the fMRI part, participants completed structural scans and thereafter a first functional scan while receiving baseline moderate and high pain stimuli. Thereafter, three scans covered the acquisition and extinction of nocebo hyperalgesia. Finally, the second learning task, exit questionnaire, and a debriefing were completed.

During the screening part (see Fig. 1), participants signed an informed consent form and completed the health, psychiatric, and MRI screening for inclusion in the study. Sensory and pain threshold levels were then tested, and pain stimuli were calibrated for each participant. The electrodes were then attached to the hand and arm and the short mock calibration of the sham electrical stimulation took place. Preconditioning was then completed. During the MRI part, participants first completed the WMS–IV and then received the oral pharmacological administration. During a 2-h waiting time for DCS to take effect, participants had a small, standardized meal, completed the psychological questionnaires, and prepared to enter the MRI scanner. Then, participants entered the scanner, completed a structural scan, and were then exposed to the baseline pain stimulations. Participants then underwent the nocebo acquisition and extinction procedures. After the end of the experiment, participants completed the second WMS–IV, were asked to answer the exit questions, and then were debriefed and reimbursed for their participation.

### Statistical analysis

#### Data screening and behavioral measures

Analyses of demographic, psychological, and behavioral measures were performed for descriptive purposes and as manipulation checks. Behavioral data were analyzed and visualized by use of *R* programming software (version 4.1.2; R Core Team, 2019), including the MASS^54^, stargazer^55^, and ggplot2^56^ packages.

The magnitude of reported nocebo hyperalgesia was measured within-subjects and was defined as the difference in pain ratings for the first nocebo trial compared to the first control trial of the extinction phase (i.e., evocation). The first evocation trials were selected to check whether significant nocebo hyperalgesia was induced, as previous studies indicate the effect to be clearest in those trials^14,57^. We also compared the average of pain ratings of the first 10 trials of extinction (i.e., evocation) for a significant nocebo response, as these were used for brain imaging analysis where more trials are required. Repeated measures ANOVAs were conducted with *trial type* as within-subjects factor with two levels (nocebo trial, control trial), as a separate model, to test whether significant nocebo hyperalgesia was induced.

The first and last pairs of trials of the extinction phase were used to calculate the magnitude of extinction of nocebo responses. The reduction of nocebo responses was measured as the change in magnitude of nocebo responses (nocebo minus control trial difference score) between the start and the end of the extinction phase. A repeated-measures ANOVA was performed with *time of measurement* (pre to post) as within-subjects factor with two levels (nocebo magnitude before extinction, nocebo magnitude after extinction).

For the purpose of a thorough exploration of the data, we additionally performed the main behavioural, pharmacological, and fMRI analyses on a group of consistent nocebo responders which was drawn based on a 3SD method adapted from previous studies^58^. To test nocebo responses, the effects of DCS, and brain activations among only those participants who were had clear and consistent nocebo responses, we drew a group including those participants that reported nocebo evocation trials within 3 SD from their mean nocebo ratings during conditioning. We identified 28 participants as consistent nocebo responders, of which 13 received placebo and 15 received DCS. The detailed results of these additional exploratory analyses are reported in Supplementary material.

Pearson correlation analyses were performed between all questionnaire data (psychological questionnaires, exit questions) and the magnitude of nocebo responses (nocebo minus control trial difference score), to establish whether these factors impacted nocebo responding. We also conducted post-hoc exploratory mediation analyses, to explore potential between-groups mediating effects of the questionnaire scores on nocebo magnitudes.

For all behavioral analyses the threshold for significance was set at *p* < 0.05 and partial eta-squared (*η*_*p*_^*2*^) was computed as a measure of effect size, with *η*_*p*_^*2*^ of 0.01 considered small, 0.06 considered medium, and 0.14 considered a large effect size^59,60^. To conduct analysis of variance (ANOVA) and correlations, potential outliers and the assumptions of normality and homogeneity were checked.

#### Pharmacological manipulation

To test the first hypothesis, that DCS would lead to larger nocebo responses than a placebo, we examined whether nocebo hyperalgesia differed between the DCS and Control groups. A 2 × 2 mixed model ANOVA was performed, with *group* as the between-subjects factor and *trial type* in evocation as within-subjects factor (first extinction nocebo trial, first extinction control trial). We also examined the reduction of nocebo magnitudes after extinction. To compare extinction between the pharmacological groups, a 2 × 2 mixed model ANOVA was performed with *group* as the between-subjects factor and *time of measurement* (pre to post) as within-subjects factor with two levels (nocebo-control trial difference *before* extinction, nocebo-control trial difference *after* extinction).

#### fMRI analyses

In preprocessing the data, anatomical scans were skull stripped with the Brain Extraction Toolbox (BET;^61^. Subsequent preprocessing was conducted in SPM12 (Wellcome Department of Cognitive Neurology, London; www.fil.ion.ucl.ac.uk/spm) running on MATLAB 2021A (MathWorks, Natick MA, USA; https://www.mathworks.com/products/matlab.htm. Functional scans were realigned to correct for motion artifacts and low frequency drift, and six motion regressors (x, y, z, pitch, roll, yaw) were obtained to account for temporally correlated head motion artefacts in the design matrix. Low frequency drift was corrected with a 128 s high pass filter. Structural scans were coregistered to the mean functional image calculated during realignment. Functional images were rigid-body coregistered to the structural scan, which was then normalized to Montreal Neurological Institute (MNI) space with 2 × 2 × 2 mm voxels. The resulting deformation field was then applied to the functional images. A Gaussian spatial smoothing kernel of 6 mm full width at half maximum was applied to the functional images. Data were visually inspected for successful co-registration. The four functional scans were preprocessed separately, then concatenated to one set of functional images per participant. Heart rate and respiratory data were preprocessed with the PhysIO toolbox^62^.

For statistical analysis, functional images were modeled on a design matrix consisting of columns for 1, Baseline moderate pain trials; 2, Baseline high pain trials; 3, Acquisition control trials; 4, Acquisition nocebo trials; 5, Evocation control trials; 6, Evocation nocebo trials; 7, Extinction control trials; 8, Extinction nocebo trials (columns 1–8 of the design matrix, each modeled with the onset and duration of the approximately 7000 ms pain stimulus); 9, Pain rating periods; 10, Control and nocebo anticipatory cues; 11, RETROICOR regressors for heart rate, respiration, and heart rate-respiration interaction^63^; 12, respiratory volume per time; 13, heart rate variability (HRV; 11–13 estimated with the PhysIO toolbox); and 14, six motion regressors for the rigid body transformation. All task regressors (1–10) were convolved with the hemodynamic response function. Data were additionally high-pass filtered with a cut-off of 128 s and corrected for temporal autocorrelation with a first-order autoregressive model.

First level analyses pertaining to our hypotheses contrasted acquisition control trials with acquisition nocebo trials, evocation control trials with evocation nocebo trials, and baseline high pain trials with evocation nocebo trials. The evocation phase consisted of the first 10 extinction trials (evocation) and exploratory analyses of the extinction phase included the remaining 18 extinction trials (labeled as exploratory because hypotheses for extinction of nocebo responses were not explicitly stated in the pre-registration of this study). Second level analyses compared these contrasts between and across pharmacological groups. Masks pertaining to a priori regions of interest (ROI) including the ventrolateral prefrontal cortex (vlPFC), dorsolateral prefrontal cortex (dlPFC), amygdala, anterior cingulate cortex (ACC), operculum, and insula were drawn from the Harvard–Oxford Atlas (HOA;^64^ in FSLeyes^65^. Masked ROI analyses were conducted separately per ROI. Statistical significance for all contrasts was corrected with a familywise error rate (FWE) correction to a p value of *pFWE* < 0.05. This was further adjusted with a Bonferroni correction per hypothesis to *pFWE* < 0.01 to correct for five ROI analyses per hypothesis, with a minimum cluster size of 10 voxels (2 mm MNI space). Imaging data visualizations were carried out using FSL^66^.

## Results

### Participants and pain reports

**Figure 2:**
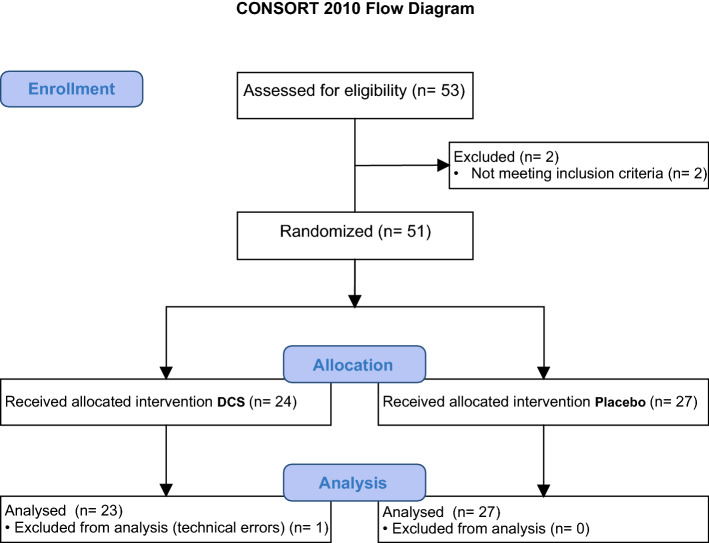
Flow-chart of participants’ movement through the trial, based on CONSORT 2010.

Fifty-three participants were enrolled in this study and 2 were excluded upon screening for inclusion, based on their medical history (Fig. 2). The data of 1 participant that completed the study were excluded due to technical errors in the experiment. A total of 50 participants (39 women) were included in the final analyses. The mean age of participants was 23 years (SD = 3.3; Table 1). Table 1 also displays the mean warmth and pain detection thresholds, mean temperatures used to induce moderate and high pain, and reported pain differences during baseline as well as nocebo acquisition and extinction. Five participants that received DCS and two that received placebo self-reported noticing mild dizziness (*n* = 2 in the DCS group), or sleepiness/tiredness (*n* = 2 in the DCS group, *n* = 1 in the placebo group).

**Table 1:** Descriptive statistics of demographics, temperatures used, pain ratings, questionnaires, and learning rates.

	Mean DCS	Mean Placebo	Mean sample	SD sample	Min sample	Median sample	Max sample
Age	22.4	23.3	22.9	3.3	18	22	35
Moderate pain used (°C)	46.9	46.7	46.8	0.6	45.0	46.9	48.0
High pain used (°C)	48.5	48.4	48.5	0.5	47.0	48.5	50.0
Warmth threshold (°C)	34.3	33.9	34.1	1.3	32.8	33.8	40.0
Pain threshold (°C)	43.8	45.1	44.5	2.6	34.8	45.1	47.2
Pre-conditioning pain difference *	3.4	3.2	3.3	0.9	1.3	3.4	5.7
Baseline pain difference *	2.6	2.3	2.4	1.3	− 0.5	2.3	6.1
Acquisition pain difference *	3.6	3.3	3.4	1.3	0.9	3.4	6.8
Extinction first trials pain diff	1.8	1.8	1.8	1.5	− 1.0	2.0	5.0
Extinction first five trials pain diff	1.2	1.1	1.1	1.1	− 0.9	0.9	3.9
Extinction final trials pain diff	0.8	0.7	0.7	0.6	− 0.3	0.5	2.2
PCS score	25.6	24.7	25.1	6.8	13.0	24.0	45.0
BVS score	16.9	17.1	17.0	5.7	6.3	17.4	31.4
STAI state score	31.3	33.9	32.7	8.7	20.0	33.3	60.0
STAI trait score	36.3	38.4	37.5	7.4	25.0	37.0	58.0
Learning^±^ score total pre-DCS	5.6	5.5	5.6	1.5	2.0	5.8	8.0
Learning^±^ score total post-DCS	5.2	5.3	5.3	1.8	0.8	5.5	8.0
Learning^±^ change pre/post-DCS	− 0.4	− 0.2	− 0.3	1.6	− 4.0	− 0.5	5.0

All temperatures are reported in degrees Celsius.

PCS, Pain Catastrophizing Scale; BVS, Body Vigilance Scale; STAI, Spielberger State-Trait Anxiety Inventory; DCS, D-cycloserine.

*Pain differences represent the mean NRS difference between nocebo/high pain trials minus control/moderate pain trials for each phase. In extinction, the first pair of trials (evocation), first 10 trials, and final 18 nocebo and control trials are reported.

^**±**^Learning was measured as a manipulation check with the Wechsler Memory Scale.

On average, participants reported that they believed the information they received during the study (M = 7.3, SD = 2.3), they were not concerned about what the researcher thought of them or changing their responses out of compliance (M = 0.6, SD = 1.1), they were focused on the heat stimuli (M = 8.2, SD = 1.2), and they noticed the increased pain association with the nocebo electrical stimuli (Mean = 9.2, SD = 1.2). We ran Pearson’s correlations between the magnitude of nocebo hyperalgesia and manipulation check exit questions and none of the responses to exit questions where significantly correlated with the magnitude of nocebo responses (all *p* > 0.05).

### Behavioral results

The regression assumptions of linearity and homogeneity were met, and behavioral data were normally distributed. No outliers were present, determined by Mahalanobis distance. Correlation analyses of psychological questionnaire scores (Table 1) did not yield significant associations with nocebo magnitude or any other pain measures (all *p* > 0.05). Results on the between-groups mediating effect of questionnaire scores on nocebo magnitudes also yielded non-significant results (for all paths *p* ≥ 0.05). Baseline and post-experimental learning rates are shown in Table 1 and indicate that WMS-IV learning rates remained stable from before to after DCS administration.

**Figure 3:**
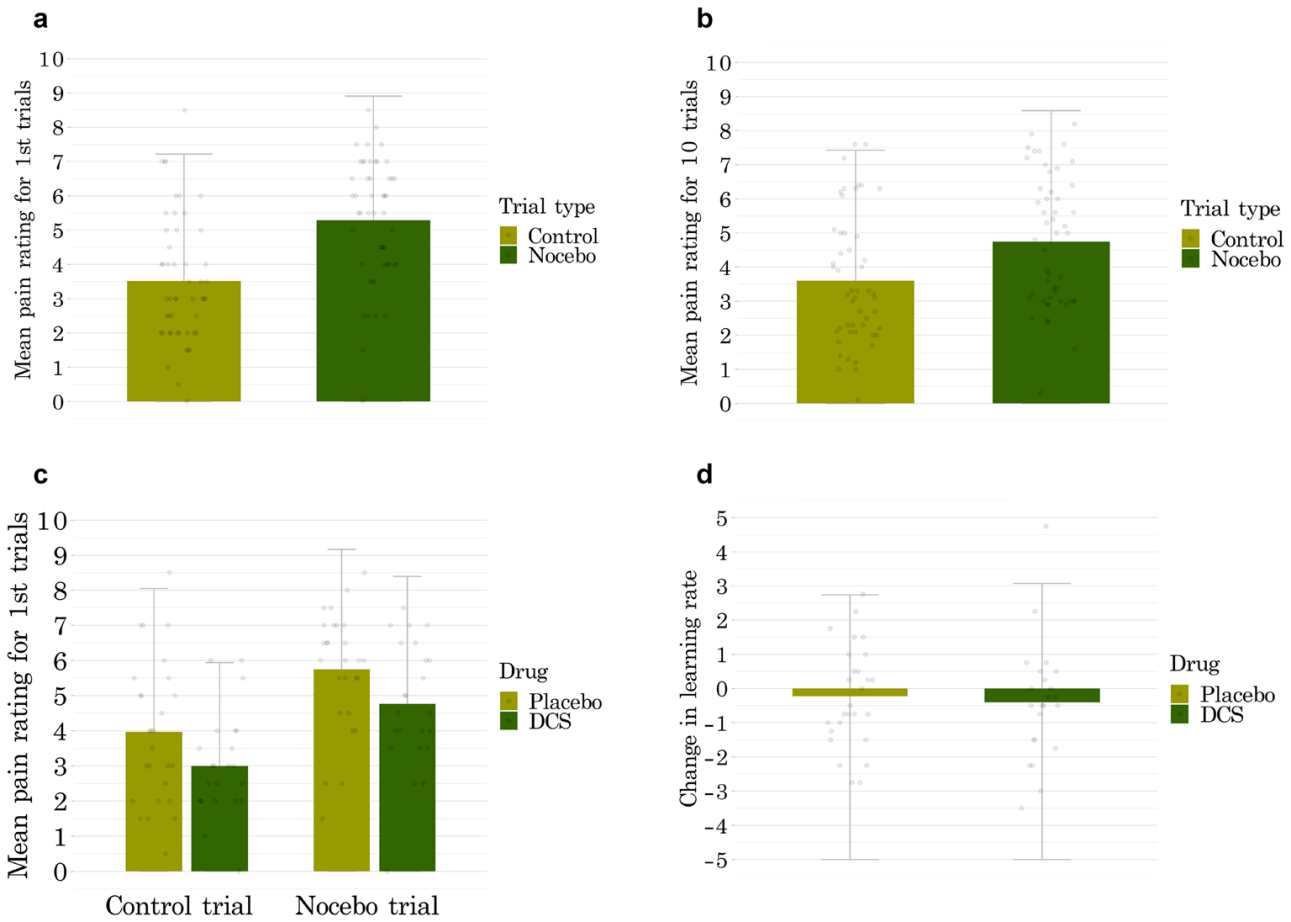
Behavioral results represented as group means and standard deviations. Dots in the figure represent individual data points. There was a significant nocebo effect in the first pair (**A**) and first 10 trials of extinction (**B**). Nocebo responses were not affected by D-cycloserine (DCS) compared to placebo (**C**). Learning rates were measured on the WMS-IV before and after DCS administration (**D**). There was only a slight non-significant reduction in learning rates of all participants, irrespective of drug group.

The nocebo manipulation successfully induced nocebo responses as measured during the first trials of extinction (Fig. 3a). Across both groups, there was a significant difference between pain reports for the first nocebo and first control trial of the extinction phase (*F* (1,49) = 73.03, *p* < 0.001, *η*_*p*_^*2*^ = 0.19) indicating the presence of nocebo hyperalgesia. We also found a significant nocebo effect in the first ten extinction trials which were used as an evocation phase for fMRI analysis (*F* (1,49) = 59.73, *p* < 0.001, *η*_*p*_^*2*^ = 0.08; Fig. 3b). Finally, there was significant extinction of nocebo responses, with the magnitude of nocebo responses being significantly lower in the last pair of (nocebo/control) extinction trials, as compared to the first pair of extinction trials (*F* (1,49) = 13.17, *p* = 0.001, *η*_*p*_^*2*^ = 0.08). When testing nocebo extinction in the last 10 (nocebo/control) extinction trials compared to the first 10 extinction trials, the extinction effect was not significant (*F* (1,49) = 0.53, *p* = 0.46, *η*_*p*_^*2*^ = 0.004).

### Pharmacological manipulation

**Figure 4:**
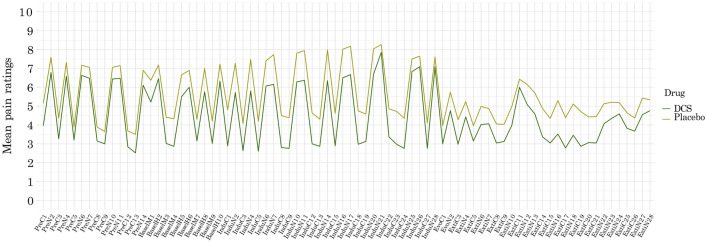
Timeseries of all trials. Mean pain ratings are shown in pre-conditioning (*Pre*) baseline (*Basel*), nocebo induction (*Indu*), and extinction (*Ext*) including the first two extinction trials (*Evocation*) where behavioral nocebo effects were measured. D-cycloserine (DCS) administration did not significantly affect the magnitude of nocebo responses. The slight group difference in ratings observed indicates a minor average difference in individually calibrated pain intensities that was already present prior to DCS administration and had no significant effects on outcomes (all *p* > 0.05).

A mixed ANOVA indicated that there was no significant interaction between drug group and the magnitude of nocebo responses based on trial type (*F* (1,48) = 0.002, *p* = 0.97, *η*_*p*_^*2*^ < 0.001; Fig. 3c). This was aligned with no increases in learning rate from pre to post drug administration in the DCS as compared to the placebo group (Figs. 3d, 4). We did not find an effect of DCS on the magnitude of extinction either, as there was no significant interaction between drug group and the reduction of nocebo responses at the end of extinction (*F* (1,48) = 0.11, *p* = 0.73, *η*_*p*_^*2*^ = 0.001).

### fMRI results

**Figure 5:**
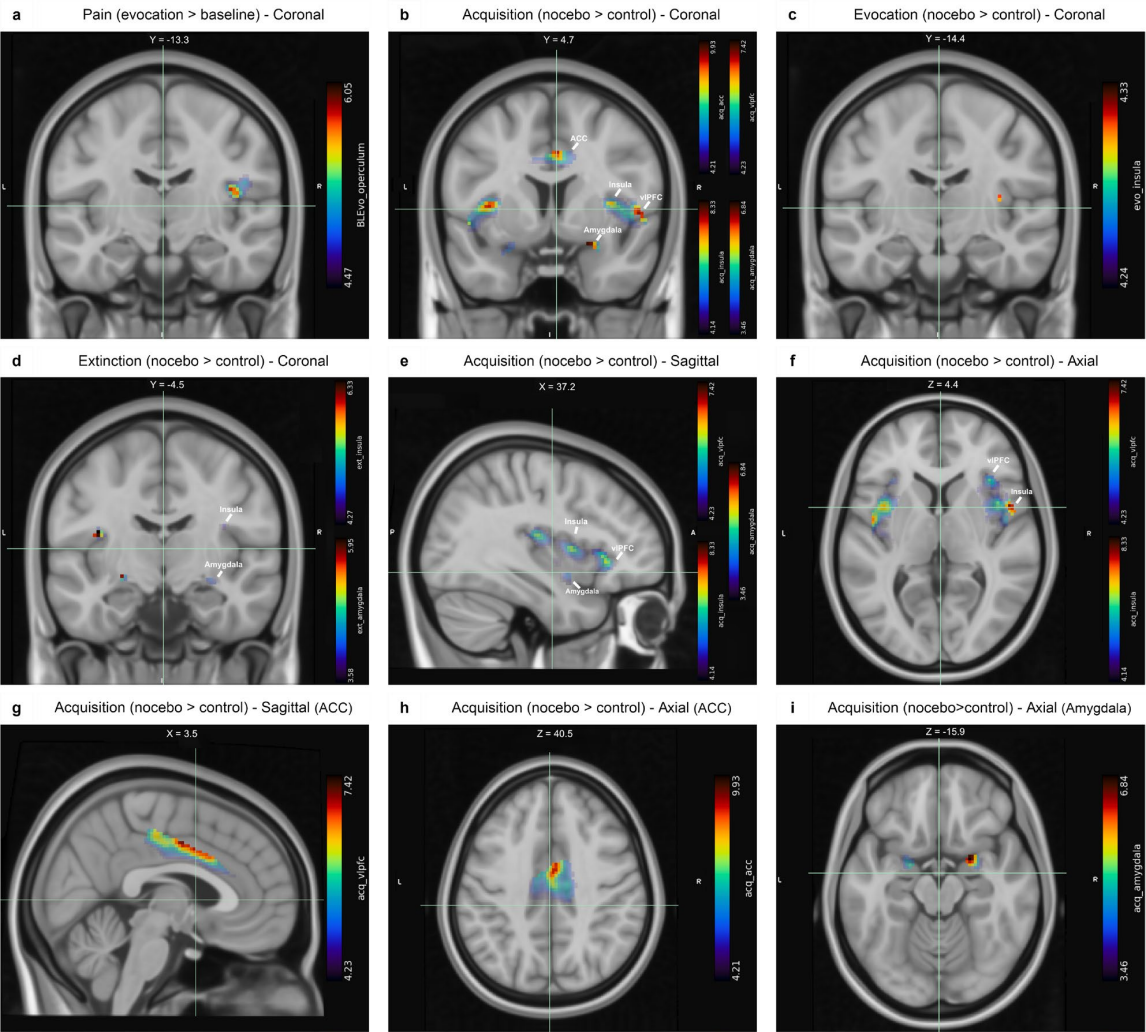
Results of the fMRI analysis. Differences in signal intensity of BOLD activations between baseline pain and nocebo-augmented increased pain responses were found in the operculum (**A**). Contrasting nocebo and control trials resulted in differential BOLD signal intensity during nocebo acquisition (**B**), evocation (first 10 extinction trials; **C**), and extinction (last 18 extinction trials; **D**). For nocebo acquisition, the significant activations in the vlPFC, insula, and amygdala in sagittal plane (**E**), the insula and amygdala in axial plane (**F**), the ACC in sagittal (**G**) and axial planes (**H**), and the amygdala in axial plane (**I**) are also shown. Location coordinates for sagittal (X), coronal (Y), and axial (Z) slices are shown above each fMRI image. Brain images are in neurological display convention.

Increased activity in evocation nocebo trials compared to baseline high pain was found in the right operculum (Table 2, Fig. 5a). During the acquisition phase, we detected an increased BOLD response during nocebo trials compared to control trials in bilateral ACC, bilateral amygdala, bilateral insula, and bilateral vlPFC (all clusters from a priori analyses are presented in Table 2, Fig. 5b, e–i).

**Table 2:** Results of a priori ROI analyses for acquisition nocebo > control, and evocation nocebo > baseline high pain contrasts.

Region (HOA mask)	MNI coordinates Left/Right (peak voxel)				t value	P value (cluster)	Voxels
**Acquisition**							
ACC	LR	2	− 6	42	9.87	< 0.001	1544
Amygdala	L	− 20	2	16	5.21	0.002	110
Amygdala	R	22	2	16	6.81	0.002	115
Insula	L	− 36	16	14	8.29	< 0.001	1467
Insula	R	36	4	8	7.05	< 0.001	1453
vlPFC	L	− 46	16	10	6.49	0.002	68
vlPFC	R	52	6	2	7.39	< 0.001	337
**Baseline-evocation**							
Operculum	R	42	− 12	14	6.04	< 0.001	173

Coordinates given in x, y, z for MNI space. T statistics calculated with df = 48, p < 0.05_FWE_.

HOA, Harvard Oxford Atlas; MNI, Montreal Neurological Institute; FWE, familywise error; vlPFC, ventrolateral prefrontal cortex; ACC, anterior cingulate cortex.

**Figure 6:**
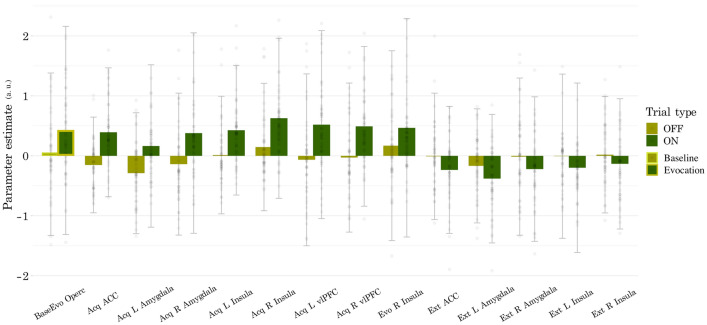
Parameter estimates are plotted for all clusters and contrasts. Raw parameter estimates were extracted using MarsBar in SPM, with no scaling applied. Parameter estimates represent the mean beta value of the entire ROI, as derived from the Harvard Oxford cortical and subcortical structures atlases. The first two, highlighted bars represent the contrast between baseline high pain stimulations and evocation phase nocebo trials when pain was augmented by the nocebo manipulation. The remaining bars represent the contrast between nocebo and control trials for each of the experimental phases.

No clusters reached the threshold for significance in the evocation (first 10 extinction trials) control and nocebo contrast initially, but notably, in exploratory analyses of the evocation phase with no minimum cluster size, we detected an increased BOLD response during nocebo trials in the left insula (all clusters from exploratory findings in Table 3, Fig. 5c), albeit this did not reach significance. Exploratory analysis of the remaining 18 extinction trials detected increased BOLD signal during nocebo trials in bilateral amygdala and insula, as well as a small, below-threshold cluster of the ACC (Table 3, Fig. 5d). Parameter estimates were computed with MarsBaR^67^ and are plotted for all clusters (Fig. 6).

**Table 3:** Results of exploratory ROI analyses for evocation (first 10 extinction trials) nocebo > control, and extinction (remaining 18 extinction trials) nocebo > control contrasts.

Region (HOA mask)	MNI coordinates Left/Right (peak voxel)				t value	P value (cluster)	Voxels
**Evocation**							
Insula	R	36	− 22	10	4.33	0.035	4
**Extinction**							
ACC	R	12	− 26	48	− 4.54	0.027	3
Amygdala	L	− 24	− 8	− 8	− 5.93	0.005	40
Amygdala	R	24	− 2	− 12	− 4.32	0.016	14
Insula	L	− 38	− 10	18	− 6.31	0.001	56
Insula	R	36	− 4	18	− 4.63	0.01	14

Coordinates given in x, y, z for MNI space. T statistics calculated with df = 48, p < 0.05_FWE_.

HOA, Harvard Oxford Atlas; MNI, Montreal Neurological Institute. FWE, familywise error; ACC, anterior cingulate cortex.

No differences between pharmacological groups were detected in any a priori ROIs, or during exploratory whole brain analyses, for the hypothesized contrasts between acquisition control and nocebo trials, evocation control and nocebo trials, or baseline high pain to evocation nocebo trials (Table 4). We further explored whether DCS would lead to differences in brain activations, as compared to placebo, only among participants that were labeled as consistent nocebo responders. We found no significant between-groups differences in any a priori ROIs (see Supplementary material).

**Table 4:** Results of between-groups a priori ROI analyses for acquisition nocebo > control, and evocation nocebo > baseline high pain contrasts.

Region (HOA Mask)	DCS parameter estimate	Placebo parameter estimate	df	*t*	*p*
**Acquisition**					
Amygdala	0.393	0.363	48	0.234	0.816
ACC	0.396	0.326	48	0.536	0.594
dlPFC	0.331	0.304	48	0.177	0.86
vlPFC	0.374	0.376	48	− 0.015	0.986
Insula	0.38	0.346	48	0.256	0.799
**Evocation**					
Amygdala	− 0.168	0.044	48	− 1.309	0.197
ACC	− 0.024	− 0.121	48	0.905	0.37
dlPFC	− 0.116	0.168	48	− 1.567	0.124
vlPFC	− 0.016	− 0.042	48	0.22	0.826
Insula	0.032	0.085	48	− 0.459	0.645
**Baseline-evocation**					
Amygdala	0.077	0.1	48	− 0.22	0.826
ACC	0.013	0.28	48	− 2.454	0.021
dlPFC	− 0.073	0.273	48	− 1.84	0.072
vlPFC	− 0.092	0.264	48	− 1.97	0.054
Insula	0.049	0.221	48	− 1.459	0.151

With a Bonferroni correction for five ROIs, significant p values are < 0.01.

HOA, Harvard Oxford Atlas; ACC, anterior cingulate cortex; dlPFC, dorsolateral prefrontal cortex; vlPFC, ventrolateral prefrontal cortex.

## Discussion

This study investigated the role of DCS on the acquisition and extinction of nocebo hyperalgesia in an fMRI study. Significant nocebo effects were induced but DCS did not influence nocebo magnitudes or brain activations, suggesting that the pharmacological manipulation did not influence learning in this nocebo paradigm. fMRI results indicated that in acquisition and extinction phases, there were significantly increased signal intensity of BOLD activations bilaterally in the amygdala, ACC, and insula, during nocebo compared to control trials. Nocebo acquisition trials also showed increased vlPFC activation. Increased opercular activation further differentiated nocebo-augmented pain aggravation from baseline high pain. These results are in line with previous nocebo studies and support the involvement of cognitive processes in nocebo hyperalgesia.

The learning paradigm induced significant nocebo responses across both groups, as was anticipated, and these effects were stronger among a group of consistent nocebo responders. The pharmacological manipulation in this study did not affect learning of verbal pairs or nocebo associations. DCS generally tended towards larger effect sizes, but still non-significant, for the group of consistent nocebo responders. Although DCS is known to impact neuroplasticity^29^ previous findings are mixed. Many studies show effects of DCS on phobia and symptoms that are known to result from aversive learning^29,34^. Yet, other studies have shown differential effects of DCS, for example facilitating procedural but not declarative learning^35^, and extinction or memory consolidation, but not necessarily acquisition of learned responses^24,27^. These differential findings could theoretically be related to the dosage used, with doses in the relevant studies mentioned above varying from 50 to 250 mg and fixed, rather than measured based on body weight. We choose a moderate dose of 80 mg. Generally, there does not seem to be an apparent dose-related efficacy of DCS, with one review of the literature reporting that neither the dose nor the time of administration had an effect on the learning outcomes^68^.

Interestingly, DCS augmentation effects have mainly been studied in phobic stimuli and for fear memory^29^. These results suggest that DCS is effective in modulating limbic NMDA circuits engaged in paradigms with a heavy fear load^69^. We did find increased amygdala activation for nocebo trials over control trials during acquisition and extinction of the nocebo effect irrespective of pharmacological group, and there seems to be some involvement of fear in nocebo^40,70,71^. Speculatively, the type of pain-learning task employed in our nocebo experiment may potentially not primarily rely on the same fear-learning circuits that DCS has been found to affect in previous studies. DCS not affecting nocebo responding may point to a potential differentiation between the specific mechanisms involved in pain-learning versus fear-learning. In other words, we speculate that learning a negative nocebo association may not involve the NMDA-mediated learning that DCS may be able to augment in more fear-specific contexts.

The amygdala has been consistently implicated in fear-learning^32,72^, but amygdala involvement may not be an essential feature or necessary prerequisite for nocebo induction. Other brain areas are shown to underlie nocebo hyperalgesia in the absence of an amygdala involvement^73–75^. Interestingly, the amygdala seems to be involved when experimental contexts or suggestions are especially negative or frightening, such as in visceral pain studies^70^ or studies of a higher threat-load that include extensive conditioning and negative suggestions^40^. In line with this, our study with pre-conditioning and negative suggestions showed increased activation of the amygdala on nocebo compared to control trials. Involvement of the amygdala in the more negative experimental contexts could suggest that fear may be a secondary modulatory factor in nocebo hyperalgesia^71,76^. Pain-related learning seems to take place on two conceptual levels. On one hand, cortical-level associative learning mechanisms may be at the core of acquiring learned pain effects. On the other hand, fear-related learning, that may take place in subcortical loops involving the amygdala, mediates pain worsening, and may be a secondary modulatory factor in pain chronification^77^.

Distinct learning mechanisms mediated via the vlPFC may also have engaged during nocebo acquisition in our study. The vlPFC is linked to learning, belief formation, and stimulus–response associations^78–82^. Neural circuits involving the vlPFC are thought to communicate through oscillations in gamma-band (60–160 Hz) frequency channels^83^. This relates to previous studies implicating gamma-band oscillations as a marker of learning in nocebo acquisition^16,84,85^. The vlPFC, as the present study also suggests, may be implicated in sensory stimuli whose properties are processed bottom-up^83^. This corresponds to participants engaging in this type of bottom-up processing of nocebo versus control stimuli, only in the acquisition phase of our experiment, before top-down processing based on learned information of nocebo associations begins taking place.

The insula and more broadly the operculum are also thought to be central cognitive features of sensory perception^86–88^. Opercular involvement is consistently found in nocebo hyperalgesia and marks mechanisms of sensory discrimination and cognitive pain modulation^37^. We also found differences in insular and opercular activations between nocebo and control stimuli. It is perhaps unsurprising that sensory modulation is involved in the acquisition of nocebo hyperalgesia. Crucially, however, we found a persistence of insular activations even when all heat administrations were equal in intensity, during extinction, albeit this was only a small cluster of activations. This may indicate that the brain continues engaging in cognitive pain discrimination during nocebo responding, when nocebo stimuli were generally perceived as more painful while all heat intensities were actually identical. Indeed, this is in line with findings of the present study indicating that nocebo responses were not completely attenuated at the end of the extinction phase. Indeed, when we tested nocebo extinction across a broader range of trials (in the last to the first 10 trials), the extinction effect was not significant, potentially reflecting that any attenuation of learned effects that does occur takes place quickly in the beginning of the extinction phase.

We also found further differences in opercular activations between evoked nocebo responses and baseline pain. Before learning took place, we administered participants with baseline high pain stimuli. Our results show increased activation of the operculum during nocebo-augmented high pain in the evocation phase, as compared to the baseline high pain stimuli. The operculum was significantly less engaged in experiencing high pain before learning took place, while increased cognitive sensory processing seems to take place when pain sensitivity is increased under nocebo hyperalgesic conditions. The consistent involvement of subregions of the operculum, ACC, and PFC in nocebo responding may underscore a primary role of cognitive and sensory integration and modulation in nocebo hyperalgesia.

A limitation of this study was in analyzing the small number of initial nocebo evocation trials, before prolonged extinction, which underpowered the evocation results. Given that extinction begins soon after the pairing of the cues and varying stimulus intensities is discontinued, only the first few extinction trials can be considered to distinctly represent nocebo evocation. One solution that would allow future studies to examine brain activity during evocation, before extinction occurs, is to reinforce nocebo associations throughout extinction. Some nocebo studies employed such continued reinforcement paradigms and achieved persistent nocebo responding that may have been less contaminated by extinction effects^37,89^. Additionally, while we conducted our power analysis for this study based on the behavioral-pharmacological primary outcome, a sample of 25 participants per group is considered a minimum required sample for fMRI analyses, and this may have led to some of the imaging results for smaller brain clusters being underpowered^90^.

This study also had other limitations that future research should address. It is important to note that the generalizability of our results may be limited, due to the recruitment of a healthy, young participant sample. Results of this study may not represent pain processing correlates in patients or individuals who have experienced severe or chronic pain, as their neurophysiology may differ from that of healthy people^91^. It is imperative for future research to replicate our findings both in patient populations and in more realistic clinical contexts.

Albeit the pharmacological manipulation using a partial NMDA receptor agonist did not affect nocebo responses, this study provided important support for the integration of cognitive-emotional and sensory processes in nocebo hyperalgesia. The acquisition of nocebo hyperalgesia was primarily characterized by increased activation in brain regions that cognitively integrate and modulate pain inputs. We showed that cognitive-emotional processing of pain signals in the operculum and ACC may integrate prior negative experiences. Understanding the intricate relationship of learning and sensory modulation in the formation of negative pain associations is highly relevant for the effective management of pain.

## Supplementary Information

Supplementary Information.

## Data Availability

The primary data files containing extracted raw data are submitted to the journal as supporting materials. The full set of materials, protocols, raw behavioral and neuroimaging files, and scripts for preprocessing and analyses will be made available via a complete publication package on the online data repository DANS (https://dans.knaw.nl/en/data-stations/life-health-and-medical-sciences/), within the first month after this study has been accepted for publication, according to Leiden University policy. The corresponding author will make available all data and materials to the journal’s Editorial Board Members and Referees immediately upon request.
