# Supplementary material for: A randomized pharmacological fMRI trial investigating d-cycloserine and brain plasticity mechanisms in learned pain responses
Source: Sci Rep. 2022 Nov 9;12:19080. doi: 10.1038/s41598-022-23769-7 (PMC9646732; doi:10.1038/s41598-022-23769-7)
Supplement: Supplementary file 1 — Supplementary Information. [file 41598_2022_23769_MOESM1_ESM.docx]

**Supplementary Material**

***Analyses for only consistent nocebo responders***

*Behavioral results*

Across both nocebo responder groups, there was a significant difference between pain reports for the first nocebo and first control trial of the extinction phase (*F* (1,27) = 42.79, *p* < 0.001, *η_p_^2^* = 0.21) indicating the presence of stronger nocebo hyperalgesic responses among consistent nocebo responders, as expected. We also found a significant nocebo effect in the first ten extinction trials which were used as an evocation phase for fMRI analysis (*F* (1,27) = 34.48, *p* < 0.001, *η_p_^2^* = 0.9). Finally, there was significant extinction of nocebo responses, with the magnitude of nocebo responses being significantly lower in the last pair of (nocebo/control) extinction trials, as compared to the first pair of extinction trials (*F* (1,27) = 15.29, *p* = 0.0005, *η_p_^2^* = 0.15). When testing nocebo extinction in the last 10 (nocebo/control) extinction trials compared to the first 10 extinction trials, the extinction effect did not reach significance (*F* (1,27) = 3.52, *p* = 0.07, *η_p_^2^* = 0.03).

*Pharmacological manipulation*

A mixed ANOVA indicated that there was no significant interaction between drug group and the magnitude of nocebo responses based on trial type for consistent nocebo responders (*F* (1,27) = 0.21, *p* = 0.65, *η_p_^2^* = 0.001). We did not find an effect of DCS on the magnitude of extinction among nocebo responders either, as there was no significant interaction between drug group and the reduction of nocebo responses at the end of extinction (*F* (1,27) = 0.23, *p* = 0.64, *η_p_^2^* = 0.003). The change in learning rates between the DCS and placebo groups (based on the Weschler Memory Scale) was not significant among consistent nocebo responders.

*fMRI results*

We further explored whether DCS would lead to differences in brain activations, as compared to placebo, only among participants that were labeled as consistent nocebo responders. We did not find significant between-groups differences when conducting the same fMRI analyses described in the methods section on only the group of consistent nocebo responders (Table S1).


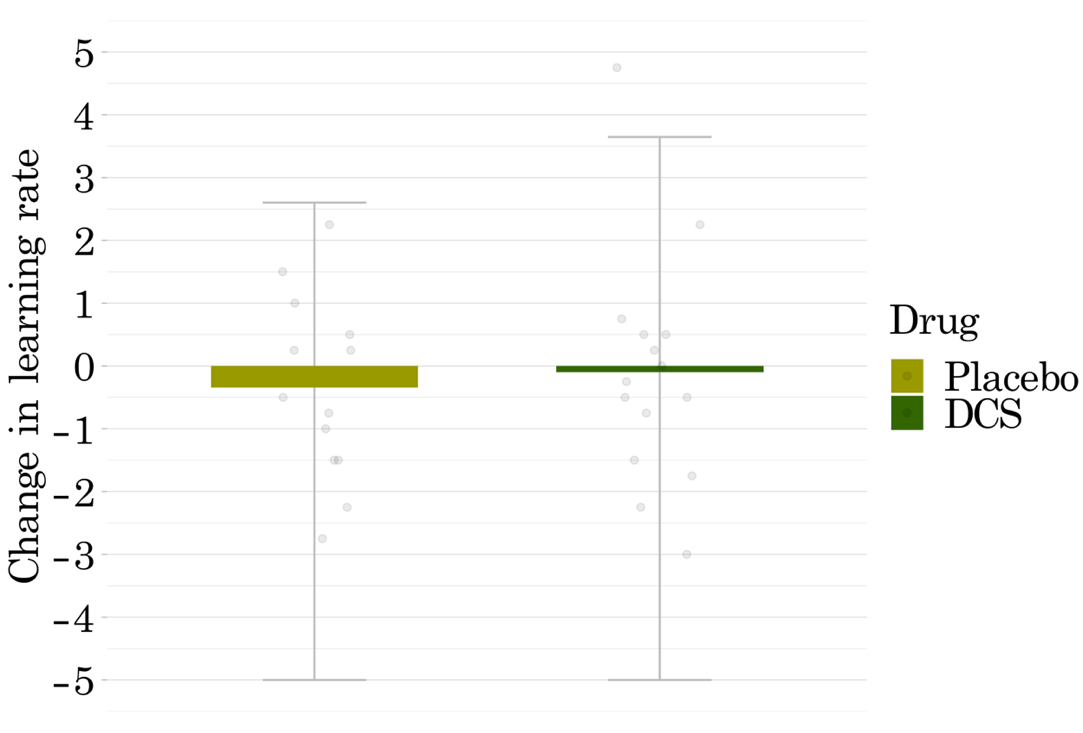


**Figure S1**. Change in learning rates from pre- to post- pharmacological administration between the DCS and placebo groups (based on the Weschler Memory Scale) was not significant among consistent nocebo responders. Dots on the graph represent individual datapoints for nocebo responders.

**Table S1**. Results of post-hoc between-groups ROI analyses for acquisition nocebo > control, and evocation nocebo > baseline high pain contrasts, using only nocebo responders.

|  | Region (HOA Mask) | DCS  parameter estimate | Placebo  parameter estimate | df | *t* | *p* |  |
| --- | --- | --- | --- | --- | --- | --- | --- |
| **Acquisition** |  |  |  |  |  |  |  |
|  | Amygdala | 0.297 | 0.199 | 26 | 0.69 | 0.496 |  |
|  | ACC | 0.355 | 0.145 | 26 | 1.259 | 0.219 |  |
|  | dlPFC | 0.237 | -0.368 | 26 | 0.433 | 0.668 |  |
|  | vlPFC | 0.3 | 0.154 | 26 | 0.895 | 0.378 |  |
|  | Insula | 0.401 | 0.318 | 26 | 0.458 | 0.651 |  |
| **Evocation** |  | | | | | | |
|  | Amygdala | -0.245 | 0.07 | 26 | -1.517 | 0.141 |  |
|  | ACC | -0.094 | 0.096 | 26 | -1.02 | 0.317 |  |
|  | dlPFC | -0.113 | 0.156 | 26 | -1.599 | 0.122 |  |
|  | vlPFC | -0.052 | 0.099 | 26 | -0.788 | 0.438 |  |
|  | Insula | 0.005 | 0.094 | 26 | -0.529 | 0.602 |  |
| **Baseline-evocation** |  | | | | | | |
|  | Amygdala | -0.061 | 0.079 | 26 | -1.104 | 0.28 |  |
|  | ACC | -0.059 | 0.19 | 26 | -1.723 | 0.097 |  |
|  | dlPFC | -0.028 | 0.494 | 26 | 2.105 | 0.045 |  |
|  | vlPFC | 0.001 | 0.174 | 26 | -1.073 | 0.293 |  |
|  | Insula | 0.013 | 0.242 | 26 | -1.521 | 0.14 |  |

*Note.* With a Bonferroni correction for five ROIs, significant p values are <.01. HOA, Harvard Oxford Atlas; ACC, anterior cingulate cortex; dlPFC, dorsolateral prefrontal cortex; vlPFC, ventrolateral prefrontal cortex.
